# Ferrocene-Functionalized 4-(2,5-Di(thiophen-2-yl)-1*H*-pyrrol-1-yl)aniline: A Novel Design in Conducting Polymer-Based Electrochemical Biosensors

**DOI:** 10.3390/s150101389

**Published:** 2015-01-13

**Authors:** Rukiye Ayranci, Dilek Odaci Demirkol, Metin Ak, Suna Timur

**Affiliations:** 1 Chemistry Department, Faculty of Art and Science, Pamukkale University, 20070 Denizli, Turkey; E-Mail: rukiye.yagiz@gmail.com; 2 Biochemistry Department, Faculty of Science, Ege University, 35100 Bornova, Izmir, Turkey; E-Mail: suna.timur@ege.edu.tr; 3 Institute on Drug Abuse, Toxicology and Pharmaceutical Science (BATI), Ege University, 35100 Bornova, Izmir, Turkey

**Keywords:** ferrocenyldithiophosphonate, conducting polymers, biosensor, glucose

## Abstract

Herein, we report a novel ferrocenyldithiophosphonate functional conducting polymer and its use as an immobilization matrix in amperometric biosensor applications. Initially, 4-(2,5-di(thiophen-2-yl)-1*H*-pyrrol-1-yl)amidoferrocenyldithiophosphonate was synthesized and copolymerized with 4-(2,5-di(thiophen-2-yl)-1*H*-pyrrol-1-yl)benzenamine at graphite electrodes. The amino groups on the polymer were utilized for covalent attachment of the enzyme glucose oxidase. Besides, ferrocene on the backbone was used as a redox mediator during the electrochemical measurements. Prior to the analytical characterization, optimization studies were carried out. The changes in current signals at +0.45 V were proportional to glucose concentration from 0.5 to 5.0 mM. Finally, the resulting biosensor was applied for glucose analysis in real samples and the data were compared with the spectrophotometric Trinder method.

## 1. Introduction

The conducting polymer field has experienced rapid and considerable advances. The latest advances in this field have led to the production of a variety of materials with a greater potential for practical applications such as batteries [1], electronic devices [2], sensors and capacitors [3,4], and electromagnetic devices (ECDs) [5,6]. New hybrid materials have been formed to improve the usage or to advance in new areas by combining substances having different properties with conductive polymers. For this reason, research studies in different branches of science have been conducted on inorganic-organic hybrid polymeric materials created by combining two building blocks with very different characteristics from each other. Incorporation of inorganic components in the organic polymeric structure has shown promising applications in various fields of chemistry such as organic synthesis, biotechnology, catalysis, electronics, and polymers [7]. Metallo-polymers, in which a metal complex is coordinated to a polymer backbone, have been well documented for applications in molecular electronics, redox catalysis and photoelectrochemistry. By incorporating metal complexes into a polymer structure, the physical and chemical properties of the resulting material may be modified by the metal complexes present, while the film-forming properties exhibited by the polymer can be retained. Advantages of metal containing polymers as electrochromic materials include facile preparative procedures, versatile tuning ability of absorption bands in the visible region associated with metal-to-ligand charge transfer and ligand-to-metal charge transfer transitions, and fast switching rates between on- and off-states by applying different electrode potentials [8].

Because of the high application potential in sensor systems, functionalization of conducting polymers (CPs) is an increasing subject. As a result of modification of conventional monomers such as pyrole, thiophene *etc.*, the usage of CP as immobilization matrix becomes more advantageous. Various strategies have been developed for the design of CP-based biosensors such as entrapment of biomolecules in the CP backbone during the electropolymerization process, adsorption or covalent binding on the surface of CP-modified support. Covalent bonds, especially crosslinks between the functional groups of biomolecules and polymer provides are the strongest immobilization [9]. Recently, Azak *et al.* synthesized N-functionalized dithienopyrroles and electropolymerized onto the graphite electrode as a novel conducting polymer matrix, then glucose oxidase (GOx) was immobilized onto the amino functional surface by means of glutaraldehyde [10]. Furthermore the conducting polymer of 2-(2,5-di(thiophen-2-yl)-1*H*-pyrrol-1-yl) acetic acid was electrochemically synthesized onto a graphite electrode by cyclic voltammetry and immobilization of GOx onto the polymer was performed through covalent binding using carbodiimide coupling method by Ekiz *et al.* [11]. In another study, 4-(2,5-di(thiophen-2-yl)-1*H*-pyrrol-1-yl) benzenamine (SNS-NH_2_) was electrodeposited onto the graphite electrode and used as an immobilization support for different biocomponents such as enzyme [12,13] and *G. oxydans* cells [14]. Recently, Oztekin *et al.* investigated direct electron transfer from GOx immobilized on polyphenanthroline-modified glassy carbon electrode [15]. Besides, Zor *et al.* reported anamperometric GOx biosensor using a 1,10-phenanthroline-5,6-dione-modified electrode [16].

Monomeric ferrocene (Fc) was initially used in electron-shuttling redox couples. The solubility of the oxidized form would inevitably be a source of long-term instability, due to loss of ferricenium ions that can diffuse away from the electrode surface, while Fc-containing conducting polymer with relatively high molecular weight can serve as stable electrically sensor for molecular recognition, as mediators in amperometric biosensors or as coatings to modify electrode surfaces. Thus, redox-active Fc-containing conducting polymers are good candidates to play a key role as multi-electron transfer mediators in electrochemical response processes of biological and industrial importance [17].

Several reports are available on first generation biosensors where conducting polymers were used. The principle of these biosensors was mainly based on the monitoring of oxygen consumption at −0.7 V (*vs.* Ag/ACl) [10–14,18]. As an alternative to first generation enzymatic biosensors the mediated biosensors have several advantages such as less susceptibility to interfering substances and less dependency on oxygen concentration during measurements [19]. Mediators can also increase current densities for substrate oxidation and decrease the operating potential. Fc derivatives have generally been used as redox mediators. Biosensing methods including Fc were previously reported by different groups. Yilmaz *et al.* synthesized chitosan–Fc hybrid through covalent modification. This redox-active hybrid was further employed as a support for immobilization of GOx and whole cells of *Gluconobacteroxydans* using glutaraldehyde on a glassy carbon electrode [20]. Then Karadag *et al.* reported an electrochemical biosensor mediated by using 6-(ferrocenyl)hexanethiol (FcSH) by construction of gold nanoparticles on the surface of polyamidoamine dendrimer (PAMAM)-modified gold electrode. GOx was used as a model enzyme and immobilized onto the gold surface, forming a self-assembled monolayer via FcSH and cysteamine [21]. Another glucose biosensor was fabricated based on a chitosan-bovine serum albumin cryogel with multiwalled carbon nanotubes (MWCNTs), Fc, and GOx [22]. Furthermore, Senel *et al.* presented a novel copolymer of Fc branched polypyrrole for the construction of an amperometric glucose biosensor [23]. In another study, pyrrole and Fc carboxylate-modified pyrrole (P(Py-FcPy)) has been polymerized electrochemically on an indium-tin-oxide (ITO) coated glass surface and GOx was entrapped during deposition for the fabrication of mediator-less electrochemical biosensor [24]. Since mediated biosensors exhibit less dependency on oxygen concentration, and less sensibility to interfering substances during measurements which are the critical points for the biosensor fabrication, combination of the conducting polymer with a well-known redox mediator is the main advantage of our system.

Both amine and Fc functionalized monomers have been used to prepare a copolymer as a mediated biosensor component. Initially, 4-(2,5-di(thiophe-2-yl)-1*H*-pyrrol-1-yl)aniline (SNS-NH_2_) and then, 4-(2,5-di(thiophen-2-yl)-1*H*-pyrrol-1-yl)amidoferrocenyldithiophosphonate (SNS-NH_2_-Fc) were synthesized and electrochemically copolymerized. The amino groups of SNS-NH_2_ were used to immobilize GOx using glutaraldehyde as the crosslinking agent. The Fc groups in SNS-NH_2_-Fc were responsible for the mediated electrode responses. Monomer feed ratio and pH of the medium were optimized and glucose analysis in real samples was carried out.SNS-NH_2_/SNS-NH_2_Fc/GOx biosensor showed good linearity and better detection limit for glucose when compared to conducting polymer-based second generation glucose biosensors in the literature.

## 2. Experimental Section

### 2.1. Chemicals

Dichloromethane (DCM), AlCl_3_, thiophene were purchased from Merck (Darmstadt, Germany). Tetrabutylammoniumhexafluorophosphate (TBAFP), acetonitrile (ACN), glacial acetic acid, succinyl chloride, HCl, sodium bicarbonate were from Aldrich (Berlin, Germany). Toluene, D-glucose, ethanol, glucose oxidase (GOx, from *Aspergillus niger* 200 U/mg), glutaraldehyde (25%) were purchased from Sigma (Berlin, Germany).

### 2.2. Instrumentation

Chronoamperometry measurements were carried out by a Radiometer electrochemical measurement unit (www.radiometer.com, Lyon, France) with three electrode systems. Palm Instruments (PalmSens, Houten, The Netherlands) and a three electrode system were used for cyclic voltammetry (CV) and chronocoulometry studies. Ag/AgCl, Pt and graphite electrodes were used as reference, counter and working electrode, respectively. Fluorescence microscopy studies were performed using an Olympus BX53F fluorescence microscope (Olympus Europa SE & CO. KG, Hamburg, Germany) equipped with a green filter, an Olympus camera, and an UplanSapo 100× apoakromat objective (Olympus Europa SE & CO. KG, Hamburg, Germany) to show fluorescence images of co-polymer before and after enzyme immobilization.

### 2.3. Synthesis of 4-(2,5-Di(thiophen-2-yl)-1H-pyrrol-1-yl)aniline and 4-(2,5-Di(thiophen-2-yl)-1H -pyrrol-1-yl)amidoferrocenyldithiophosphonate

**Scheme 1.**
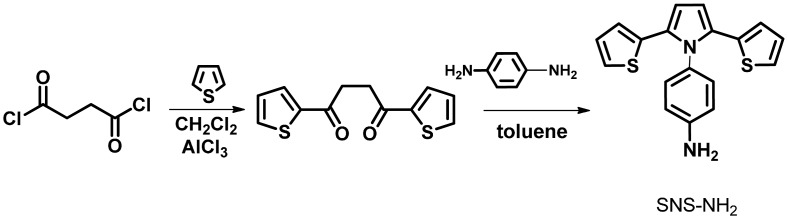
Synthesis of SNS-NH_2_.

To synthesize 4-(2,5-di(thiophen-2-yl)-1*H*-pyrrol-1-yl)aniline (SNS-NH_2_), 1,4-bis-2-thienylbutane-1,4-dione (0.004 mol), *p*-phenylenediamine (0.006 mol), glacial acetic acid (5 mL) and toluene (50 mL) were mixed (Scheme 1). The mixture was refluxed for 4 days with a Dean-Stark trap and flash chromatography was applied to the resultant product. 4-(2,5-di(thiophen-2-yl)-1*H*-pyrrol-1-yl) aniline (SNS-NH_2_). The monomer was synthesized in a yield of %72 [25].

**Scheme 2.**
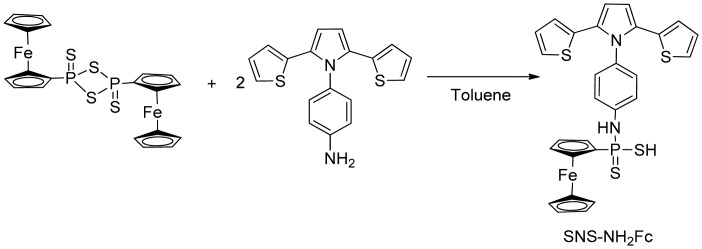
Synthesis of SNS-NH_2_-Fc.

The monomer SNS-NH_2_-Fc was synthesized by the reaction with (SNS-NH_2_) and ferrocenedithiophosphanate. Briefly, [FcP(=S)(μ-S)rlsqb;_2_ (0.21 g, 0.39 mmol) and 4-(2,5-di(thiophen-2-yl)-1*H*-pyrrol-1yl) aniline (0.25 g, 0.78 mmol) in toluene (25 mL) was stirred (Scheme 2). With time the solid was dissolved by heating. The reaction mixture was filtered and the solution was allowed to stand at −18 °C. The resulting yellow-orange crystalline product was filtered and dried under vacuum. 4-(2,5-di(thiophen-2-yl)-1*H*-pyrrol-1-yl)amido ferrocenyl dithiophosphonate (SNS-NH_2_-Fc) was obtained in 60% yield [26].

### 2.4. Biosensor Preparation

**Scheme 3.**
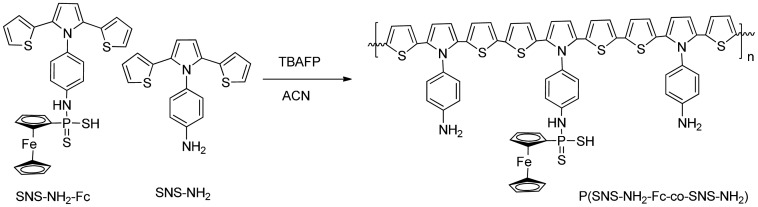
Schematic representation of the electrochemical copolymerization of P(SNS-NH_2_-Fc-*co*-SNS-NH_2_).

Before each deposition step, graphite rods were polished with emery paper and rinsed several times with deionized water. A mixture of SNS-NH_2_ (1.0 mg/mL) and SNS-NH_2_-Fc (4.0 mg/mL) were electrochemically polymerized on the graphite electrode in 0.1 M TBAFP/ACN electrolyte/solvent system at room ambient conditions via scanning the potential between −0.5 V and +1.5 V at a scan rate of 100 mVs^−1^ using cyclic voltammetry (Scheme 3). After completion of electropolymerization, the polymer coated surface of the electrode was rinsed with deionized water to get rid of the impurities.

To immobilize GOx on the conducting polymer-modified graphite electrodes, 1.0 mg GOx (which equals to 21.2 U) in 10 μL potassium phosphate buffer solution (50 mM, pH 7.5) and 1.0% glutaraldehyde (10 μL, (50 mM, pH 7.5)) were spread over the surface and allowed to dry at room temperature for 2 h. Before electrochemical measurements, it was rinsed with deionized water to remove unbound enzyme molecules and reagents.

### 2.5. Measurements

Chronoamperometric measurements were performed in a reaction cell containing 10 mL working buffer solution (50 mM, pH 7.5) under gently stirring. During the measurements, the current changes due to the enzymatic activity before and after glucose addition were followed at +0.45 V. Change of current as the response of the biosensor was recorded in each measurement. The working solution was refreshed after each measurement.

### 2.6. Sample Application

The SNS-NH_2_/SNS-NH_2_-Fc/GOx biosensor was tested with real samples (Coke and Fizzy). Samples were degassed and diluted with working buffer and then injected into the working buffer instead of glucose as a substrate. Calibration curves for glucose were used to determine the glucose contents in measured samples. The glucose detection in real samples was also calculated using a commercial enzyme assay kit based on spectrophotometric Trinder reaction (Glucose MR, Cat. No. 1129010, Cromatest, Barcelona, Spain) as the reference method and results were compared with those obtained with the constructed biosensors. In the Trinder reaction, the glucose is oxidized to D-gluconate by GOx with the formation of hydrogen peroxide. In the presence of peroxidase, a mixture of phenol and 4-aminoantipyrine (4-AAP) is oxidized by hydrogen peroxide to form a red quinoneimine dye proportional to the glucose concentration in the sample [27].

## 3. Results and Discussion

### 3.1. Characterization of the Monomers

**Figure 1.**
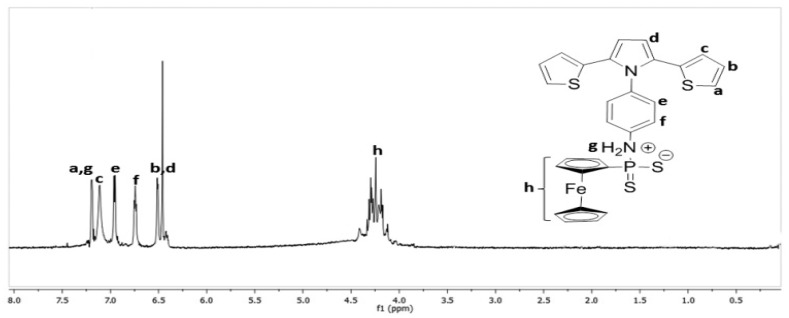
^1^H-NMR spectrum of (SNS-NH_2_-Fc).

The synthesis and characterization of a novel conductive inorganic-organic hybrid monomer was described here. Fc moiety as an electroactive group acts as a redox mediator that allows mediated electrochemical sensing at +0.45 V. Various redox mediators such as Fc as well as a range of organic dyes have been described in the literature [16]. However, the absence of functional groups such as primary amine (-NH_2_) or carboxyl (-COOH) groups restricts the possibility of covalent binding of the enzyme to the surface. To eliminate this disadvantage, SNS-NH_2_-Fc was copolymerized with SNS-NH_2_. Initially, the monomers were synthesized and characterized. To show the presence of Fc moieties in the monomer backbone, NMR spectra were recorded. The ^1^H-NMR spectrum strongly supports the proposed monomer structure. Figure 1 shows the ^1^H-NMR spectrum of (SNS-NH_2_-Fc) when CDCl_3_ used as solvent. Chemical shift values are as follows: ^1^H NMR C_18_H_14_N_2_S_2_ (400 MHz, CDCl_3_) δH ppm: 4.00–4.50 (m, 9H, Hh), 6.46 (s, 2H, Hb), 6.51 (dd, *J* = 2.91 Hz, 2H, Hd), 6.73 (dd, *J* = 4.30 Hz, 2H, Hf), 6.75 (d, *J* = 4.06 Hz, 2H, He), 6.96 (dd, *J* = 4.92 Hz, 2H, Hc), 7.17 (d, *J* = 6.03 Hz, 2H, Hb), 7.19 (s, 4H, Ha, Hg).

**Figure 2.**
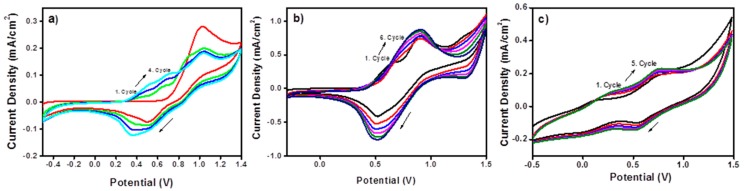
Cyclic voltammograms of (**a**) (SNS-NH_2_) and (**b**) (SNS-NH_2_-Fc) and (**c**) (SNS-NH_2_)/(SNS-NH_2_-Fc) (1:4; w/w) in ACN/TBAPF solvent-electrolyte system at a scan rate of 0.25 V/s on ITO.

Electropolymerization of monomers was carried out by cyclic voltammetry. While Pt and silver wires were used as a counter and reference electrodes, TBAFP/ACN was utilized as the electrolyte/solvent system. Cyclic voltammogram of (SNS-NH_2_) indicated one oxidation peak at +1.0 V and one reduction peak at +0.51 V owing to monomer oxidation and polymer reduction. Other cycles have two consecutive oxidation peaks at +0.54 and +0.71 V and two consecutive reduction peaks at +0.35 and +0.51 V owing to oxidation and reduction of polymer when the range between −0.5 and +1.4 V was scanned (Figure 2a). However, the first cycle of the cyclic voltammogram of SNS-NH_2_-Fc indicated two oxidation peaks at +0.65 that is Fc oxidation and +0.9 V and consecutive reduction peaks at +0.7 and +0.52 V (Figure 2b). A potential lower than 1.3 V promoted lower adherence of deposit and a greenish cloud is formed around the electrode due to the partial dissolution in the medium of neutral linear oligomers. Potentials above +1.3 V, seem to be most useful in obtaining insoluble, adherent and electroactive films while the greenish cloud is also formed. Only under these conditions the monomer gives a polymer film, which is subsequently oxidized at the same potential to produce polarons balanced with TBAFP. Hence, in order to obtain uniform, adherent deposits of oxidized P(SNS-NH_2_-Fc), the potential was swept between −0.5 V and +1.5 V. As the number of cycle increases, there is an increase in the intensity of the current. This is due to an increase of the total amount of electroactive polymer deposited onto the electrode. To investigate the copolymer formation, we performed CV studies in the presence of SNS-NH_2_ and SNS-NH_2_-Fc (1:4 w,w) under the same experimental conditions. As the first cycle of monomer CVs exhibited, the onset oxidation potentials of SNS-NH_2_ and SNS-NH_2_-Fc were located at about 0.62 and 0.52 V, respectively. The small distinction between the onset oxidation potentials of the two monomers indicates that the copolymerization of the monomers in the binary solvent system is feasible. As the CV continued, the conducting polymer film was formed on the working electrode surface. The small increase of the redox wave current densities implied that the amount of the polymer deposited on the working electrode surface was increasing gradually. There was a drastic change in the voltammogram as both the current increase between consecutive cycles and the oxidation potential of the material were different than those of SNS-NH_2_ and SNS-NH_2_-Fc, which in fact, could be interpreted as the formation of copolymer (Figure 2c).

### 3.2. Biosensing Applications

Herein, a novel monomer, 4-(2,5-di(thiophen-2-yl)-1*H*-pyrrol-1-yl)amido ferrocenyldithiophosphonate (SNS-NH_2_-Fc) was successfully synthesized and electrochemically co-polymerized with 4-(2,5-di(thiophen-2-yl)-1*H*-pyrrol-1-yl)aniline (SNS-NH_2_). SNS-NH_2_-Fc and SNS-NH_2_ were used as a mediator and a matrix for enzyme immobilization because of the presence of free amino groups, respectively. GOx was immobilized on the co-polymer modified graphite electrode surfaces *via* crosslinking procedures using glutaraldehyde to obtain a stable immobilization. Optimum pH and the most proper monomer feed ratio for the prepared biosensor were found. SNS-NH_2_/SNS-NH_2_-Fc/GOx matrix was characterized as an electrochemical glucose biosensor and applied for glucose detection in real samples.

**Figure 3.**
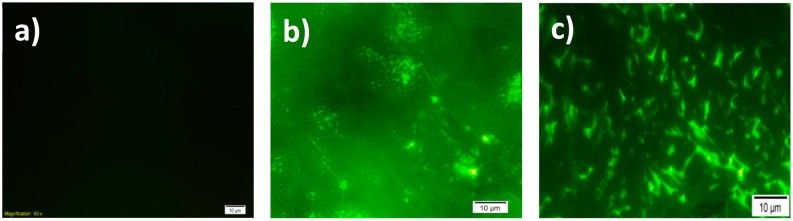
Fluorescence images of ITO surface (**a**) and SNS-NH_2_/SNS-NH_2_-Fc before (**b**) and after (**c**) GOx immobilization; 100× magnification (scale bars = 10 μm).

In order to characterize the surface after co-polymer modification and immobilization of GOx, images of fluorescence microscope were recorded (Figure 3) to investigate any possible changes in surface morphology after GOx immobilization. Fluorescence images can be used to show the success of the immobilization on the conducting polymer-based electrodes [10,28–30] and they are generally similar to SEM images, making them preferred as an alternative of SEM [9]. SNS-NH_2_/SNS-NH_2_-Fc/GOx modified surfaces were monitored by fluorescence microscopy after formation on the ITO glass. Figure 3a shows the co-polymer-modified surfaces before the immobilization of GOx and SNS-NH_2_/SNS-NH_2_-Fc/GOx modified surfaces can be seen in Figure 3b. GOx (β-D-glucose: oxygen 1-oxidoreductase, EC 1.1.3.4) contains two identical subunits, which are flavine adenine dinucleotide (FAD) cofactor bound to the polypeptide chains [31,32]. Due to the FAD centers, GOx and polymeric matrix exhibit fluorescent properties; hence it is possible to monitor biomolecule immobilization *via* fluorescence microscopy.

**Figure 4.**
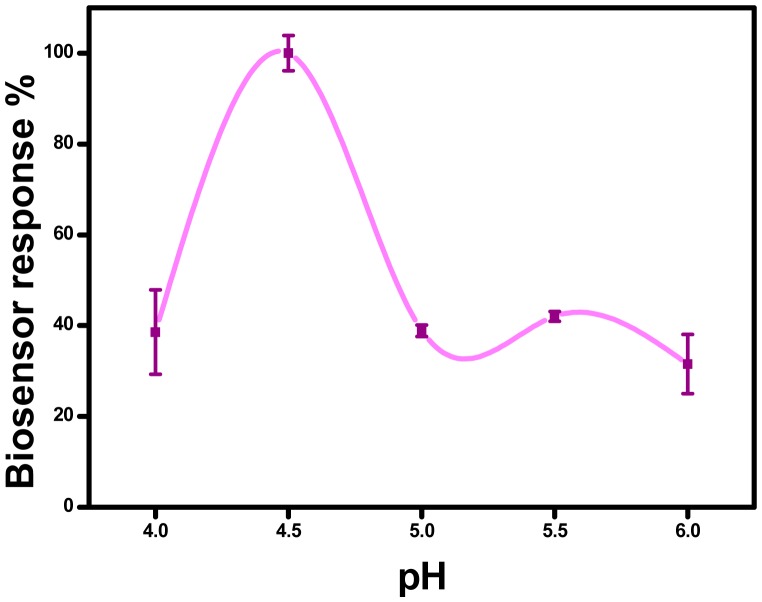
Effect of pH (in sodium acetate buffer, 50 mM, at pH 4.0–5.5 and in sodium phosphate buffer at pH 6.0; SNS-NH_2_/SNS-NH_2_-Fc: 0.5/4.5 mg; +0.45 V, [Glucose]: 5.0 mM). Error bars show standard deviation.

Working at correct pH values is important for biosensors to follow enzyme activity or the biosensor response to substrates. In biosensor fabrication, the nature of the immobilization matrix,which includes positively or negatively charged groups, affects the pH values that are used as a working buffer, so trials for the identification of working buffer pH are a critical point for any analysis with enzyme biosensors. To investigate the influence of working buffers (pH), measurements were carried out in sodium acetate (50 mM; pH 4.0–5.5) and sodium phosphate buffers (50 mM; pH 6.0). The dependency of biosensor response on pH is depicted in Figure 4. It was found that the current responses increased with the increase of pH up to 4.5, then a decrease in the current responses was observed. Thus, pH 4.5; 50 mM sodium acetate buffer was chosen as the optimum pH for the further experiments. On the other hand, Free GOx has a broad activity range of pH 4.0–7.0 (as quoted by the manufacturer, Sigma-Aldrich, St. Louis, MO, USA) and the obtained optimum pH value is in agreement with the required pH values reported for the free enzyme.

**Figure 5.**
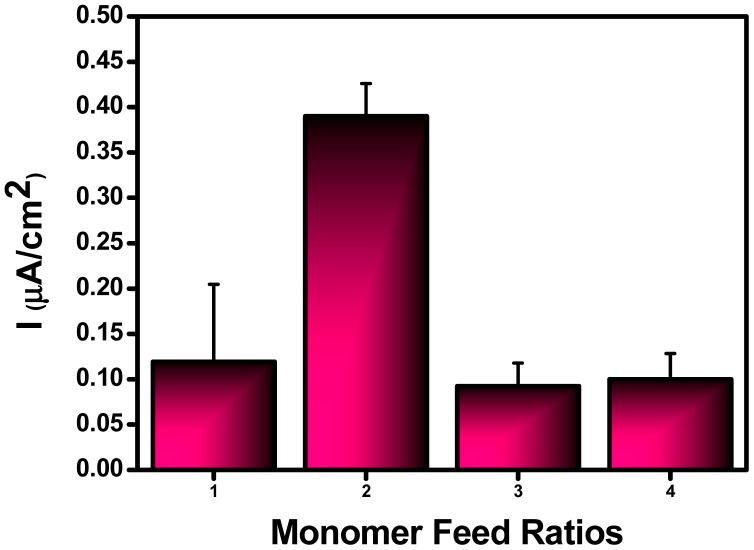
Effect of monomer feed ratio on the biosensor response (SNS-NH_2_/SNS-NH_2_-Fc ratios: (1): 0.5/4.5 mg; (2): 1.0/4.0 mg; (3): 2.0/3.0 mg and (4): 5.0/5.0 mg: in sodium acetate buffer, 50 mM, pH 4.5, +0.45 V, [Glucose]: 5.0 mM). Error bars show standard deviation.

To test the effect of monomer ratio to biosensor response, four different electrodes were prepared using different SNS-NH_2_/SNS-NH_2_-Fc ratios: 0.5/4.5 mg; 1.0/4.0 mg; 2.0/3.0 mg and 5.0/5.0 mg. After GOx immobilization on the co-polymer modified surfaces, biosensor responses were followed for a glucose standard solution. The highest currents were obtained by the biosensor with 1.0/4.0 mg SNS-NH_2_/SNS-NH_2_-Fc in the matrix (Figure 5).

**Figure 6.**
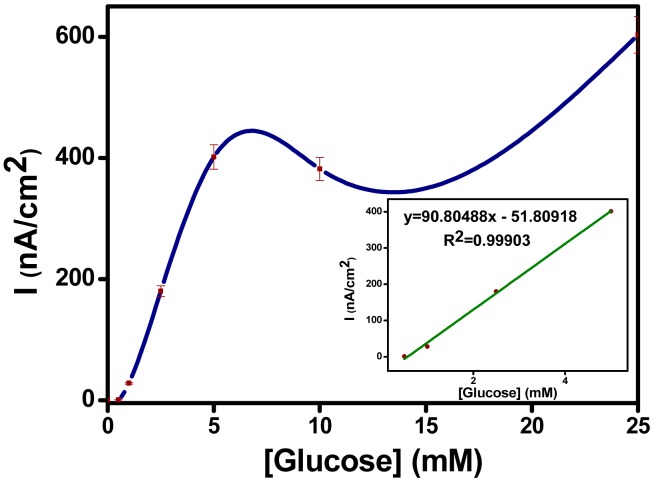
Calibration curve of SNS-NH_2_/SNS-NH_2_-Fc/GOx enzyme electrode (in sodium acetate buffer, 50 mM, pH 4.5, +0.45 V).

The current density dependence on substrate concentration has been examined and the calibration curves have been plotted for the enzyme electrode prepared by SNS-NH_2_/SNS-NH_2_Fc/GOx. Amperometric responses of the enzyme electrodes have been obtained by measuring the current densities (in μA/cm^2^) at +0.45 V (Figure 6). Linearity was obtained from 0.5 to 5.0 mM glucose with LOD of 0.18 mM (according to S/N = 3). A calibration graph between biosensor response (y) and substrate concentration (x) was defined with the equation of y = 0.908x − 0.052 (R^2^ = 0.999). The obtained SNS-NH_2_/SNS-NH_2_Fc/GOx biosensor showed good linearity and better detection limit for glucose when compared to second generation conducting polymer-based glucose biosensors described in the literature. The comparison of the analytical performance of conducting polymer-based second generation glucose biosensors is shown in Table 1.

**Figure 7.**
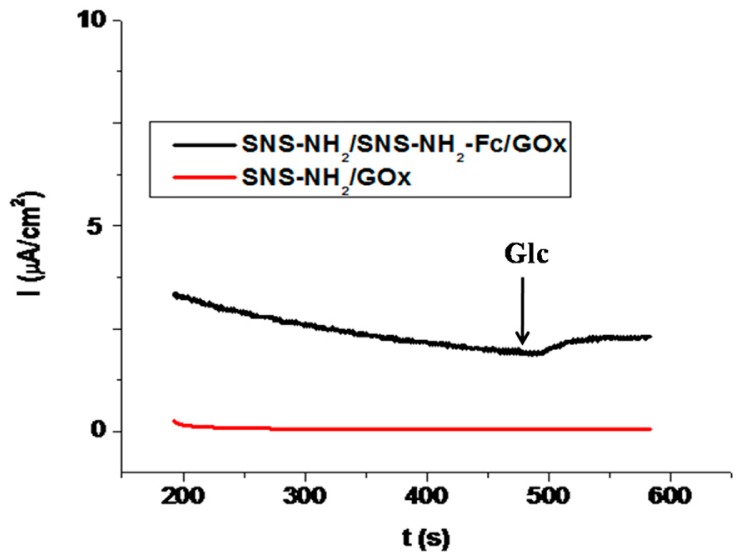
A typical chronoamperometric signal of SNS-NH_2_/SNS-NH_2_-Fc/GOx and SNS-NH_2_-Fc/GOx biosensor after glucose addition (in sodium acetate buffer, 50 mM, pH 4.5, +0.45 V, [Glucose]: 5.0 mM).

Chronoamperometric signals of the SNS-NH_2_/SNS-NH_2_-Fc/GOx and SNS-NH_2_-Fc/GOx biosensor are shown in Figure 7. To test the repeatability of the SNS-NH_2_/SNS-NH_2_-Fc/GOx biosensor, successive measurements of glucose (n = 5) were carried out and standard deviation and variation coefficient (%) were calculated as ±0.103 mM and 5.0%, respectively. Table 2 summarizes the comparison of the analytical characteristics ofSNS-NH_2_/SNS-NH_2_-Fc/GOx biosensor with others reported in literature. According to the working conditions such as type of reference and working electrode, enzyme active centre, enzyme immobilization technique and whether bound or unbound of mediator, the applied potential to obtain biosensor response in second generation amperometric biosensors which use Fc as a mediator can be varied [20,21,33–35]. In our case, it can be said that the interaction of Fcon the polymer surface with the active center of the enzyme could be performed efficiently and followed properly at the applied potential of +0.45 V/Ag/AgCl.

The SNS-NH_2_/SNS-NH_2_-Fc/GOx biosensor was applied to the determination of glucose content in some commercial beverages such as coke and fizzy and the obtained results were compared with the results obtained from spectrophotometric method. The comparison of results was summarized in Table 3.

## 4. Conclusions

In conclusion, a new strategy for the preparation of a mediated biosensor based on ferrocenyldithiophosphonate-functionalized conducting polymer was shown here. The Fc-containing monomer is a very attractive compound, because it plays a role as a mediator and can be easily electropolymerized together with unmodified polymer (SNS-NH_2_). Thus, an additional mediator for the electrochemical response is not required. Moreover, the method described here overcomes the problems associated with the first generation biosensors such as the oxygen deficiency during the enzymatic reaction, *etc.* Finally the system was successfully applied for glucose analysis in real matrices.

## Figures and Tables

**Table 1.** Some features of conducting polymer-based second generation glucose biosensors in the literature.

**Electrode**	**Conducting Polymer/Enzyme**	**Principle of Detection (Working Potential)**	**Linearity for Glucose**	**Km**	**Reference**
ITO	P(Py-FcPy)/GOx	+0.175 V (*vs.* Ag/AgCl)	2.8–16.8 mM	1.6 mM	[24]
GCE	Py/Py-CO2H/Py-Fc/GOx	+0.38 V (*vs.* Ag/AgCl)	1.0–4.0 mM	4.73 mM	[23]
GE	SNS-NH_2_/SNS-NH_2_Fc/GOx	+0.45 (*vs.* Ag/AgCl)	0.5–5.0 mM	3.86 mM	This study

GCE: Glassy carbon electrode; GE: Graphite electrode; ITO: Indium thinoxide; P(Py-FcPy):Co-polymer of pyrrole and ferrocenecarboxylate modified pyrrole; Py/Py-CO_2_H/Py-Fc: Pyrrole/N-(3-(1H-pyrrol-1-yl)ethyl)ferrocenecarboxate; ferrocene (Fc).

**Table 2.** Some features of glucose biosensors which prepared using immobilized Fc in the literature.

**Electrode**	**Immobilization Matrix**	**Biological Material**	**Working Potential (V)**	**Linear Range for Glucose (mM)**	**Response Time (s)**	**Km**	**Reference**
GCE	CHIT–Fc	GOx	+0.35V (*vs.* Ag/AgCl)	2.0–16.0	20	-	[20]
GCE	CHIT–Fc	*G.oxydans*	+0.35V (*vs.* Ag/AgCl)	1.5–25.0	70	-	[20]
Au	AuNP/(FcSH+Cyst)/PAMAM	GOx	+0.35 V (*vs.* Ag/AgCl)	1.0–5.0	-	-	[21]
Au	MWCNTs/Chi-BSA-Fc	GOx	+0.175 V (*vs.* Ag/AgCl)	0.01–30.0 mM	150	1.5 mM	[22]
GE	SNS-NH_2_/SNS-NH_2_Fc	GOx	+0.45 (*vs.* Ag/AgCl)	0.5–5.0 mM	100	3.86 mM	This study

GCE: Glassy carbon electrodes; CHIT–Fc: Chitosan–ferrocene; Au: Gold electrode; AuNP/(FcSH+Cyst)/PAMAM: Gold nanoparticles/6-(Ferrocenyl)hexanethiol/cysteamine/polyamidoamine dendrimer; GE: Graphite electrode; SNS-NH_2_/SNS-NH_2_Fc: 4-(2,5-di(thiophen-2-yl)-1*H*-pyrrol-1-yl)amido ferrocenyldithiophosphonate *co*. 4-(2,5-di(thiophen-2-yl)-1*H*-pyrrol-1 yl) aniline; ITO: Indium tin oxide; MWCNTs/Chi-BSA-Fc: Chitosan-bovine serum albumin (Chi-BSA) cryogel with incorporated multiwalled carbon nanotubes (MWCNTs), ferrocene (Fc).

**Table 3.** Results for glucose analysis in real samples by of SNS-NH_2_/SNS-NH_2_-Fc/GOx biosensor and spectrophotometric method.

**Sample**	Glucose (g/L) *		**Recovery (%)**
	**SNS-NH_2_/SNS-NH_2_-Fc/GOx**	**Spectrophotometric**	
Coke	69.1 ± 1.9	70.0 ± 3.0	99
Fizzy	55.7 ± 1.7	54.2 ± 9.3	103

* Data were calculated as the average of 3–4 trials ± S.D.
